# Plant a Vine

**Published:** 1867-04

**Authors:** 


### PLANT A VINE.

Horace Greeley has offered a large premium in money to the township which shall first have two grape vines growing on every homestead; ten years ago, we suggested in this Journal, and that even in towns and cities, every owner of a lot should have a few vines growing as a means of profitable exercise, recreation and botanical study in the cultivation, and of enjoyment in the fruitage,the shade of the leaves and the ornamentation. The Iona and Israella Vines seem to have a multitude of admirers, these may be had by addressing C. W. Grant, Iona, near Peekskill, Westchester county, New York, who "offers choice buds of Iona and Israella for propagation,at the following cash rates, namely. In lots of 20 and upward, post-paid, by mail, ten cents each. Per 100, post-paid, by mail, $7.50. Per 1000, delivered to the Express, $50 to $60. A still greater reduction on large lots.

"Those who consult the Price-List will find the prices very low for the present cost of production, while the quality of the plants is very high; and I do not hesitate to claim for them not only unequaled quality, but unequaled cheapness, when quality is considered. The constant testimony of the results of former years coming to me from all quarters affirms their excellence in all localities, and my present stock is of quality that challenges competition. Sample vines of Iona and Israella will be sent in lots of six, by mail, post-paid, on receipt of price at dozen rate.

"I especially desire all large purchasers to visit and carefully inspect the vines, and note their quality and manner of production. I have many thousands of them grown in open ground, the soil being of only fair vineyard fertility. The plants were grown at such large distances apart that the roots and leaves were allowed abundant room for full development, "stocky growth," and hardy ripening.

"My stock also comprises many thousands grown in large pots, which are worth much more for hardy vigor and abundant early bearing. For relative value of plants, see Pamphlet and Manuel, where the subject is thoroughly treated.

"Remittances by check or draft are preferred. Small amounts may be sent by money orders on New York post-office. Where these are not obtainable, national currency may be sent at my risk, if inclosed in presence of postmaster where mailed. To collections C. O. D. of less than $50, will be added return charges. All vines sold for cash. By the new Postage law, vine packages of less than four pounds are admitted by the mail. Ten cents per single vine, or sixty cents per dozen, in addition to price for one year old vines, will secure them to the purchaser by mail post-paid. *Manual of the Vine*, comprising Illustrated and Descriptive Catalogues, sent for 50c.

